# 2D porous hexaniobate-bismuth vanadate hybrid photocatalyst for photodegradation of aquatic refractory pollutants

**DOI:** 10.1016/j.heliyon.2024.e39235

**Published:** 2024-10-10

**Authors:** Shirin P. Kulkarni, Vikas V. Magdum, Yogesh M. Chitare, Dhanaji B. Malavekar, Jin H. Kim, Sultan Alshehri, Jayavant L. Gunjakar, Shashikant P. Patole

**Affiliations:** a Centre for Interdisciplinary Research, D. Y. Patil Education Society (Deemed to Be University), Kolhapur, 416 006, MS, India; b Optoelectronic Convergence Research Centre, Department of Materials Science and Engineering, Chonnam National University, Gwangju, 61186, South Korea; c Department of Pharmaceutics, College of Pharmacy, King Saud University, Riyadh, 11451, Saudi Arabia; d Department of Physics, Khalifa University of Science and Technology, AbuDhabi, 127788, United Arab Emirates

**Keywords:** Metal-semiconductor hybrid, Photocatalyst, Methylene blue, Rhodamine-B, Tetracycline hydrochloride antibiotic

## Abstract

Metal oxide semiconductors are highly promising due to their excellent photocatalytic performance in the photodegradation of industrial waste containing refractory chemical compounds. A hybrid structure with other semiconductors provides improved photocatalytic performance. In this work, porous and two-dimensional (2D) hexaniobate-bismuth vanadate (Nb_6_-BiVO_4_) Z-scheme hybrid photocatalysts are synthesized by chemical solution growth (CSG) of BiVO_4_ over electrophoretically deposited Nb_6_ thin films. The structural and morphological analysis of Nb_6_-BiVO_4_ hybrid thin films evidenced the well-crystalline uniform growth of monoclinic scheelite BiVO_4_ over lamellar Nb_6_ nanosheets. The Nb_6_-BiVO_4_ hybrid thin films exhibit a highly porous randomly aggregated nanosheet network, creating the house-of-cards type morphology. The Nb_6_-BiVO_4_ hybrid thin films display a strong visible light absorption with band gap energy of 2.29 eV and highly quenched photoluminescence signal, indicating their visible light harvesting nature and intimate electronic coupling between hybridized species beneficial for photocatalytic applications. The visible-light-driven photodegradation performance of methylene blue (MB), rhodamine-B (Rh-B) dyes, and tetracycline hydrochloride (TC) antibiotic over Nb_6_-BiVO_4_ hybrid are studied. The best optimized Nb_6_-BiVO_4_ thin film shows superior photocatalytic activity for photodegradation of MB, Rh-B dyes, and TC antibiotic with photodegradation rates of 87.3, 92.8, and 64.7 %, respectively, exceptionally higher than that of pristine BiVO_4_. Furthermore, the mineralization study of Nb_6_-BiVO_4_ thin film is conducted using chemical oxygen demand (COD) analysis. The optimized Nb_6_-BiVO_4_ thin film shows superior percentage COD removal of 83.33, 85.42, and 61.36 % for MB, Rh-B dyes and TC antibiotic, respectively. The present results highlight the expediency of hybridization in enhancing the photocatalytic activity of pristine BiVO_4_ by minimizing its charge recombination rate and improving chemical stability.

## Highlights

• Synthesis of highly porous 2D hexaniobate-bismuth vanadate hybrid photocatalysts.
• Investigation of photo-physicochemical features with comprehensive characterization tools.
• A strong visible-light absorption and highly quenched photoluminescence.
• Effective electronic coupling between Nb_6_ and BiVO_4_ with easy charge transport.
• Efficient photodegradation of methylene blue dyes, rhodamine-B dyes, and tetracycline hydrochloride antibiotic.

## Graphical abstract

**Figure:**
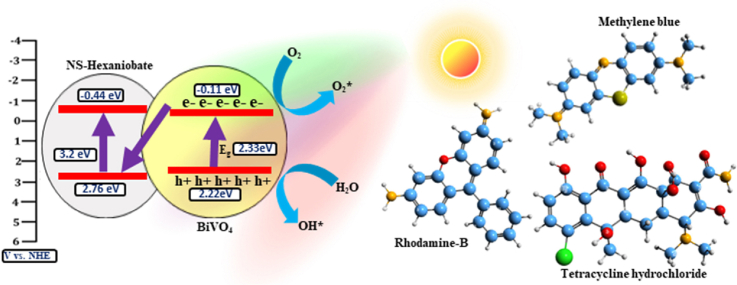

## 1 Environmental implications

Increasing water pollution has been considered one of the significant problems for aquatic life as well as human beings, causing cancers and various biological disorders. There are several reasons for aquatic pollution, out of which the toxification of water by refractory chemicals from textile industries and residual antibiotics coming from the extensive use of antibiotics to treat different diseases have a major contribution. Hence, efforts have been made to develop advanced oxidation processes to degrade these hard-to-treat pollutants. Photocatalysis is one of the advanced oxidation processes that can efficiently degrade recalcitrant molecules using solar energy in the presence of semiconductor photocatalysts. In the present work, we have developed a novel photocatalyst, i.e., 2D Porous hexaniobate-bismuth vanadate (Nb_6_-BiVO_4_) nanohybrid for the photodegradation of organic aquatic pollutants. Additionally, the photo-physicochemical features of Nb_6_-BiVO_4_ photocatalyst are investigated in detail to probe their superior functionalities in photocatalytic multitargeted degradation applications. The effect of hybridization of BiVO_4_ with Nb_6_ on the visible-light-induced photocatalytic degradation of methylene blue, rhodamine-B dyes and tetracycline hydrochloride antibiotic is explored together with their structural and physicochemical features. The present findings vividly highlight the usefulness of Nb_6_-BiVO_4_ photocatalyst for environmental remedy by degrading refractory chemicals causing aquatic pollution.

## 2 Introduction

Increasing aquatic pollution has been considered one of the significant problems for marine life as well as human beings. There are several reasons for water pollution, out of which the toxification of water by refractory chemical molecules has a major contribution [[1], [2], [3]]. Various organic dyes are primarily used in textile industries to color fabrics. Synthetic dyes are also widely used in many fields of advanced technology, namely in several textile, paper, leather tanning, food processing, plastics, cosmetics, rubber, printing and dye manufacturing industries [4,5]. Their discharge into the hydrosphere causes a significant source of pollution due to their refractory nature. Many of these dyes have adverse effects on the environment. Some of them are carcinogenic and give rise to various biological disorders [6].

In addition, the extensive use of antibiotics to treat different diseases causes environmental pollution and risks to human health. When the drug is processed through the human body, its residues come in contact with the environment [7,8]. Thus, removing such antibiotics from the environment becomes a challenging issue. Hence, efforts have been made to develop advanced oxidation processes to degrade these hard-to-treat pollutants. Photocatalysis is one of the advanced oxidation processes that can efficiently degrade recalcitrant molecules using solar energy in the presence of semiconductor photocatalysts [9,10]. Photocatalysis is one of the advanced oxidation processes that can efficiently degrade recalcitrant molecules using solar energy in the presence of semiconductor photocatalysts. The heterogeneous semiconducting photocatalytic degradation is mainly based on the photoinduced charge separation and transport to the semiconductor surface upon exposure to light irradiations. Electron-hole pairs are generated when photons with energy equal to or greater than the band gap of the semiconductor are incident on it. The generated electrons and holes move toward the surface of the semiconductor to carry redox reactions. The holes react with H_2_O molecules to form hydroxyl radicals (OH∗). On the other hand, the reaction of electrons with O_2_ molecules produces OH∗ radicals via the formation of superoxide radicals (∗O_2_^−^), hydroperoxyl radicals (HOO∗) and H_2_O_2_. Generated OH∗ radicals are strong oxidizing agent which can degrade adsorbed target molecules [[11], [12], [13]]. Numerous semiconductor materials like CoS-supported ZnAl_2_O_4_ catalyst, fluorinated TiO_2_-clay nanocomposite, TiO_2_-clay nanocomposite, TiO_2_@phosphogypsum composite have been exploited for visible-light active photocatalysis in view of efficient use of the solar spectrum [[14], [15], [16], [17]]. However, most photocatalysts ever developed are inappropriate for visible-light-photocatalysis because of their wide band gap energy, poor photostability and unsuitable band positions for reducing protons and oxidizing oxide ions [18,19].

Consequently, efforts are made to couple two types of photocatalysts with suitable band positions. Such a hybridization strategy can enable effective electron-hole pair separation via intimate electronic coupling between the coupled semiconductors [20,21]. Recently, two-dimensional (2D) metal oxide nanosheets (MONs) have emerged as macromolecules for synthesizing hybrid materials. The 2D MONs demonstrated excellent photocatalytic performance when hybridized with narrow band gap semiconductors [22,23].

Particularly, the 2D nanosheets of hexaniobate (Nb_6_) evoke high research interest as macromolecules for the hybrid photocatalysts due to their highly anisotropic 2D morphology, ultrathin thickness, high surface area, electrostatic surface charged, high mechanical flexibility, suitable band positions for the reduction of hydroxide ions as well as oxidation of water molecules and high chemical stability [[24], [25], [26]]. In addition, narrow band gap BiVO_4_ exhibits superior characteristics like high photostability, high optical absorption coefficient, resistance to photo-corrosion, suitable band positions for water splitting and low environmental toxicity [27]. Therefore, hybridization between BiVO_4_ and 2D Nb_6_ can enable expanded surface area, high photo-conductivity, superior visible light harvesting ability and improved electronic coupling useful for visible-light-induced photocatalytic multitargeted degradation. Recently researched thin films of Nb_6_ nanosheets are rarely reported. Consequently, Nb_6_-based hybrid thin films are also rarely reported. Although few reports are available on synthesizing bulk Nb-BiVO_4_-based photocatalysts, we are unaware of any prior report on synthesizing 2D hybrid thin film photocatalysts based on layered Nb_6_ and BiVO_4_ using the present method. Moreover, this method can be easily extended to develop various types of other 2D nanosheet-based nanohybrid thin films.

As compared to others, the chemical solution growth (CSG) method for the deposition of BiVO_4_ layers on the surface of Nb_6_ thin films can be treated as an effective method for the synthesis of Nb_6_-BiVO_4_ hybrid thin films because the CSG approach is simple, inexpensive and appropriate for large-area deposition carried out in air at low temperatures with a variety of substrates. It offers numerous advantages, like easy control over film thickness, preparative parameters and growth. It has a flexible chemical composition that allows the tuning of physicochemical properties of the deposited material.

In the present work, we developed a novel approach for producing highly porous Nb_6_-BiVO_4_ hybrid thin films by depositing BiVO_4_ over electrophoretically deposited (EPD) Nb_6_ thin films. Additionally, the physicochemical features of Nb_6_-BiVO_4_ hybrid thin films are investigated to probe their superior functionalities in photocatalytic multitargeted degradation applications. The effect of hybridization of BiVO_4_ with Nb_6_ on the visible-light-induced photocatalytic degradation of methylene blue (MB), rhodamine-B (Rh-B) dyes and tetracycline hydrochloride (TC) antibiotic is explored together with their structural and physicochemical features.

## 3 Experimental section

### 3.1 Materials

Niobium pentoxide (Nb_2_O_5_), potassium carbonate (K_2_CO_3_), bismuth nitrate pentahydrate (Bi(NO_3_)_3_.5H_2_O), sodium metavanadate (NaVO_3_), EDTA disodium salt (C_10_H_14_N_2_Na_2_O_8_), sodium hydroxide (NaOH), hydrochloric acid (HCl), tetra butyl ammonium hydroxide (TBAOH) and absolute ethanol (C_2_H_5_OH) were purchased from Sigma-Aldrich and used as received. Indium-doped tin oxide (ITO) coated glass substrates were used for deposition.

### 3.2 Experimental details

The Nb_6_-BiVO_4_ hybrid thin films were synthesized by deposition of BiVO_4_ on Nb_6_ thin films. The CSG and EPD were used to deposit BiVO_4_ and Nb_6_ thin films, respectively. The synthesis of Nb_6_-BiVO_4_ hybrid thin films is comprised of the following steps.

**Fig. 1:**
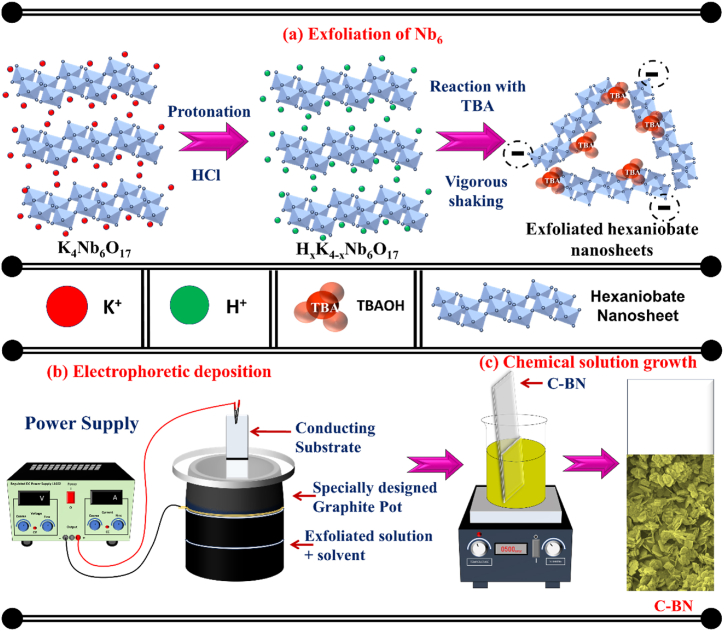
Schematic model of **(a)** exfoliation of Nb_6_, **(b)** EPD of Nb_6_ thin films and **(c)** CSG method for the deposition of Nb_6_-BiVO_4_ hybrid thin films.
(b) *Synthesis of pristine Nb*_*6*_*thin films:* Well-cleaned ITO-coated glass substrates were used to synthesize Nb_6_ thin films. The ITO-coated glass substrates were cleaned using ethanol, DDW and acetone in an ultrasonic bath. The well-cleaned ITO-coated glass substrates were stored in deionized water before the deposition to avoid contamination.

(a) *Synthesis of pristine Nb*_*6*_*:* The wide band gap and chemically stable Nb_6_ were synthesized from their host crystals of potassium hexaniobate (K_4_Nb_6_O_17_) by ion exchange and exfoliation process [28]. The host crystals of potassium hexaniobate were synthesized by solid-state reaction of an intimate mixture of K_2_CO_3_ and Nb_2_O_5_ (molar ratio of 2:3) at 1050 °C (24 h). The corresponding protonic hexaniobate (H_x_K_4-x_Nb_6_O_17_) was prepared by reaction of potassium hexaniobate powder with an aqueous solution of 1 M HCl at ambient temperature for four days. The HCl solution was exchanged with a fresh one daily throughout this proton exchange process. Afterward, the protonated solid was centrifuged, thoroughly washed with double distilled water (DDW) and air-dried to eliminate the acidic residue. The layered proton-exchanged hexaniobate was delaminated into individual Nb_6_ by intercalating TBA molecules into the interlayer space of protonic hexaniobate. For this, the weighted amount of protonic hexaniobate was reacted with an aqueous solution of TBAOH. The solution was shaken vigorously for two days, which produced the colloidal suspension of exfoliated Nb_6_. A schematic representation of the intercalation-exfoliation protocol for synthesizing Nb_6_ is shown in Fig. 1. The Nb_6_ synthesis conditions are given in Table S1 in electronic supplementary information (ESI).

Initially, the colloidal suspension of Nb_6_ (with pH of 12–12.5) was dialyzed for 14 h by using a dialysis membrane (Dialysis Membrane-135, Av. Flat width-33.12 mm, Av. diameter-23.8 mm, Capacity approx.-4.45 ml cm^−1^) in DDW to reach a pH of 8. The Nb_6_ thin films were obtained from the dialyzed Nb_6_ colloidal suspension by EPD method. The dialyzed Nb_6_ colloidal suspension (5 ml) and absolute ethanol (15 ml) were mixed in the 1:3 vol proportion for the EPD. The Nb_6_ thin films were deposited on ITO-coated glass substrates using a specially designed cylindrical-shaped graphite EPD cell by applying the potential of 15 V DC across the ITO and graphite cell electrodes. The graphite cell and ITO substrate can act as a cathode and anode, respectively. The deposition time of EPD was adjusted to a range of 5–15 min. The obtained Nb_6_ thin films were annealed at 230 °C for 1 h in the air to improve adherence and remove TBA content. The annealed Nb_6_ thin films deposited at 5, 10 and 15-min deposition times are denoted as Nb-1, Nb-2 and Nb-3, respectively.

(c) *Synthesis of Nb*_*6*_*-BiVO*_*4*_*hybrid thin films:* BiVO_4_ thin films were deposited using the CSG method on electrophoretically deposited Nb_6_ thin films. The chemical bath for the deposition of BiVO_4_ comprised 20 ml aqueous solution of 25 mM Bi(NO_3_)_3_·5H_2_O complexed with EDTA disodium salt (25 mM) under constant stirring [29]. The aqueous 20 ml stock solution of 25 mM NaVO_3_ was prepared as another precursor. The CSG bath was obtained by mixing vanadium stock solution into the bismuth complex under constant stirring. The final pH of the bath was adjusted to 5.5 using an aqueous solution of 1 M NaOH. Afterward, the bath was maintained at 85 °C under constant stirring for 1 h to obtain a transparent pale-yellow color solution.

The obtained solution was cooled to room temperature and used as a deposition solution. The Nb_6_-coated ITO substrates (Nb-1, Nb-2 and Nb-3) were immersed vertically in the above deposition bath, and the whole bath was kept in the water bath at a constant temperature of 85 °C for 10 h. After 10 h, the direct growth of BiVO_4_ on Nb_6_ thin films was observed. Subsequently, the Nb_6_-BiVO_4_-coated ITO substrates were removed from the bath, washed with DDW and air-dried. The schematic representation of the Nb_6_-BiVO_4_ thin film deposition process is shown in Fig. 1. The as-deposited Nb_6_-BiVO_4_ hybrid thin films were annealed in a muffle furnace at 400 °C for 2 h. The annealed Nb_6_-BiVO_4_ hybrid thin films deposited on Nb-1, Nb-2 and Nb-3 thin films are denoted as C-BN1, C-BN2 and C-BN3, respectively.

### 3.3 Materials characterizations

The crystal structures of pristine Nb_6_, BiVO_4_ and Nb_6_-BiVO_4_ hybrid thin films were studied with X-ray diffraction (XRD) analysis using Rigaku miniflex 600 Diffractometer (Cu k_α_ radiation: λ = 1.54 Å). The chemical bonding features of pristine materials in comparison with Nb_6_-BiVO_4_ hybrids were probed with Fourier transform infrared (FT-IR) spectra recorded on αT Bruker spectrometer. The chemical bonding of present hybrids was further explored by micro-Raman spectroscopy collected on FLEX G spectrometer (Tokyo Instrument, Japan) having an excitation wavelength of 532 nm. The oxidation states of elements present on the surface of stated materials were investigated with X-ray photoelectron spectroscopy (XPS) recorded on Thermo Scientific using Al K_α_ (1486.6 eV) X-ray source. The crystal morphology and spatial elemental distribution of the present hybrids were scrutinized with Field emission scanning electron microscopy (FESEM) analysis using JEOL JSM-7900 F electron microscope equipped with energy-dispersive spectroscopy (EDS)-elemental mapping analysis. A JEOL JEM 2100 plus, Japan (Acceleration voltage: 200 kV) transmission electron microscope (TEM) is used to obtain TEM images of the Nb_6_-BiVO_4_ hybrid. The optical properties and band structure of the hybrids were investigated with ultraviolet–visible diffuse reflectance spectroscopy (UV–vis DRS) using Jasco spectrometer. The photoluminescence (PL) spectra were obtained by using Fluoromax-4 (HORIBA instruments) spectrometer.

### 3.4 Photocatalytic measurements

The photocatalytic performance of pristine Nb_6_, BiVO_4_ and Nb_6_-BiVO_4_ hybrid thin films was evaluated for the photodegradation of MB, Rh-B dyes and TC antibiotic under visible light irradiations (light intensity = 100 mW cm^−2^). In the actual experiment, the pristine Nb_6_ or BiVO_4_ or Nb_6_-BiVO_4_ hybrid thin film (area: 1 cm^2^) was placed vertically in the quartz photoreactor filled with 3 ml of dye/antibiotic solution (concentration: 50 μM). This solution was equilibrated with the target dye/antibiotic molecules for 30 min in the dark to examine the adsorption-desorption equilibrium of the dye/antibiotic molecules on the photocatalyst surface. The light irradiations obtained from the 35 W xenon lamp were simulated with an AM 1.5 G, infrared (IR) and 420 nm optical cut-off filters. The calibrated light was focused on the quartz photoreactor. During the experiment, the Nb_6_ or BiVO_4_ or Nb_6_-BiVO_4_ hybrid thin film was removed at particular time periods, and the change in concentration of dye/antibiotic solution was analyzed by using the UV–vis spectrophotometer (Carry 60 UV–vis: Agilent Technology). The change in concentration of MB, Rh-B dyes and TC antibiotic was detected by measurement of absorbance at characteristic wavelengths of 664, 553 and 357 nm, respectively. The photocatalytic degradation performance of pristine Nb_6_, BiVO_4_ and Nb_6_-BiVO_4_ hybrid thin films was estimated from the analysis of UV–vis absorption spectra by using the following equation,

(1) $$\text{Percentage}\;\text{degradation}=\frac{C_{0}-C_{t}}{C_{0}}\times 100$$

here, $C_{0}$ and $C_{t}$ represents the absorption at initial time and at time t, respectively.

Moreover, the pseudo-first-order kinetic models are used to study photocatalytic degradation kinetics. The following equation describes a pseudo-first-order reaction,

(2) $$\ln\left(\frac{C_{0}}{C_{t}}\right)=kt$$

here, k, $C_{0}$ and $C_{t}$ represents the rate constant, absorption at initial time and at time t, respectively.

Further, the photostability of optimized thin film for photocatalytic degradation of MB and Rh-B dyes is estimated over the course of five consecutive degradation cycles. The optimized thin film is separated from the dye (MB/Rh-B) solution, washed with DDW, dried at room temperature, and used again for subsequent cycles.

The efficiency of photodegradation was further evaluated in terms of mineralization study using chemical oxygen demand (COD) analysis using the following equation,

(3a) $$\%\;\text{COD}\;\text{redution}=\frac{{COD}_{0}-{COD}_{t}}{{COD}_{0}}\times 100$$

COD_t_ and COD_0_ correspond to COD values at time t and initial conditions, respectively.

## 4 Results and discussions

### 4.1 XRD, FTIR and Raman spectroscopy

**Fig. 2:**
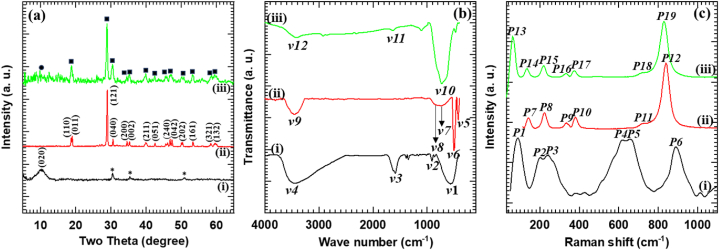
(a) XRD patterns, **(b)** FTIR spectra, and **(c)** Raman spectra of (i) Nb_6_, (ii) BiVO_4_ and (iii) C-BN2 hybrid thin films. In **(a)** asterisk, circle and squares denote the Bragg reflections of ITO, Nb_6_ and monoclinic scheelite-type BiVO_4_, respectively.

The crystallographic properties of the synthesized materials were probed by using XRD analysis. The XRD patterns of Nb_6_-BiVO_4_ hybrid thin film (C-BN2) compared with the pristine Nb_6_ and BiVO_4_ thin films are shown in Fig. 2(a). As represented in Fig. S1 (ESI), the potassium hexaniobate (K_4_Nb_6_O_17_) displays a highly intense series of (020), (040), (220), (002), (0 10 0), (063) and (400) diffraction peaks matching with the highly crystalline well-ordered layered orthorhombic structure (JCPDS: 76–0977) [30]. Its protonated product (H_x_K_4-x_Nb_6_O_17_) (Fig. S1 (ESI)) also shows an intense series of (020), (040), (002), (0 10 0) and (400) diffraction peaks. Additionally, minor signature (hkl) peaks (140), (041), (220), (240) and (063) matching with the in-plane host structure are discernible, demonstrating that the in-plane host structure remains intact after the proton-exchange process [[30], [31], [32], [33]]. The XRD pattern of pristine Nb_6_ thin film shows a high-intensity broad (020) diffraction peak corresponding to the well-ordered stacking of lamellar Nb_6_ with the orthorhombic structure having an interplanar spacing of 0.87 nm [30]. Additionally, the diffraction peaks denoted by the asterisk ‘∗’ match with the ITO substrate. Remarkably, no other peak corresponding to the different phases of Nb_2_O_5_ is present, signifying the high thermal stability of Nb_6_ thin films. The pristine CSG deposited BiVO_4_ thin film shows typical Braggs reflections that match the monoclinic scheelite structured BiVO_4_ (I2/a) (JCPDS no.: 14–0688). Interestingly, the apparent peak splitting is observed at 2θ equal to 18.5°, 35° and 46°, clearly indicating the formation of BiVO_4_ in monoclinic scheelite structure [34]. On the contrary, the C-BN2 hybrid thin film exhibits diffraction peaks corresponding to well-ordered lamellar Nb_6_ (denoted by circles) and monoclinic scheelite-type BiVO_4_ (denoted by squares). It shows a broad (020) diffraction peak matching with the well-ordered stacking of lamellar Nb_6_. It also shows typical Braggs reflections and characteristic peak splitting (18.5°, 35° and 46°) matching the monoclinic scheelite BiVO_4_. Present XRD results related to the Nb_6_ and BiVO_4_ clearly signify the growth of BiVO_4_ over EPD-deposited Nb_6_ thin films.

The chemical bonding nature and microscopic structural properties of Nb_6_-BiVO_4_ hybrids compared to the pristine Nb_6_ and BiVO_4_ thin films were investigated with FTIR spectroscopy, as shown in Fig. 2(b). The FTIR spectra of pristine Nb_6_ show absorption peaks *v*1 (576 cm^−1^) and *v*2 (901.25 cm^−1^) corresponding to the stretching vibrations of the terminal Nb-O and asymmetric stretching vibrations of the bridge Nb-O in central NbO_6_ octahedron, respectively [[35], [36], [37], [38]]. The absorption peaks at *v*3 (1626.25 cm^−1^) and *v*4 (3449.66 cm^−1^) correspond to the bending vibrations of water molecules and stretching vibrations of hydroxyl groups, respectively [39]. The pristine BiVO_4_ displays sharp absorption peaks *v*5 (413 cm^−1^) and *v*6 (482 cm^−1^) linked with the bending vibrations of Bi-O and the stretching vibrations of VO_4_^3−^, respectively [40,41]. The absorption peaks *v*7 (741 cm^−1^) and *v*8 (829 cm^−1^) are assigned to the asymmetric and symmetric stretching vibrations of VO_4_^3−^, respectively [42]. The absorption peak *v*9 (3451.15 cm^−1^) is associated with the stretching vibrations of hydroxyl groups [39]. On the other hand, the Nb_6_-BiVO_4_ hybrid shows absorption peaks *v*10 (733.75 cm^−1^), *v*11 (1626.25 cm^−1^) and *v*12 (3444.55 cm^−1^) related to the Nb_6_ and BiVO_4_. The high-intensity broad absorption peak *v*10 originated from the superposition of metal-oxygen peaks of stretching vibrations of the terminal Nb-O in central NbO_6_ octahedron, asymmetric stretching vibrations of VO_4_^3−^ and symmetric stretching vibrations of VO_4_^3−^ [35,42,43]. The absorption peaks *v*11 and *v*12 are ascribed to the bending vibrations of water molecules and stretching vibrations of hydroxyl groups, respectively [39]. The present FTIR results indicate characteristic IR features related to Nb_6_ and BiVO_4_.

The chemical bonding of Nb_6_-BiVO_4_ hybrids, as compared to pristine BiVO_4_ and Nb_6_, is further probed by micro-Raman analysis. As shown in Fig. 2(c), the Nb_6_ thin film shows a sharp Raman peak around P_1_ (92.33 cm^−1^) assigned to the internal bending modes of O-Nb-O groups [44]. The Raman peaks present at P_2_ (219.55 cm^−1^) and P_3_ (250.43 cm^−1^) are ascribed to the lattice vibration mode of highly distorted NbO_6_ octahedra and Nb-O-Nb angular deformation modes, respectively [36,45]. The Raman peaks centered within the frequency range of 500–700 cm^−1^, P_4_ (616.71 cm^−1^) and P_5_ (657.73 cm^−1^) are attributed to the stretching of longer Nb-O bonds [44]. The Raman peak at higher frequency P_6_ (895.86 cm^−1^) corresponds to the Nb-O terminal stretching mode of highly distorted NbO_6_ octahedra [45]. The pristine BiVO_4_ shows Raman peaks at P_7_ (131 cm^−1^) and P_8_ (212 cm^−1^), which are ascribed to the external modes (rotation or translation) of BiVO_4_ [46,47]. The Raman peaks present at P_9_ (325 cm^−1^) and P_10_ (367 cm^−1^) are assigned to the asymmetric (B_g_ symmetry) and symmetric (A_g_ symmetry) bending modes of the VO_4_ tetrahedron, respectively [48]. The sharp and highly intense Raman peak centered around P_12_ (826 cm^−1^) along with the shoulder at P_11_ (710 cm^−1^) are attributed to the symmetric and asymmetric (V-O) stretching modes of BiVO_4_, respectively [[46], [47], [48]]. On the other hand, Nb_6_-BiVO_4_ hybrid displays the Raman peaks attributed to the Nb_6_ and BiVO_4_. The sharp and intense Raman peak around P_13_ (92.33 cm^−1^) is assigned to the internal bending modes of O-Nb-O groups [44]. The Raman peaks P_14_ (131 cm^−1^) and P_15_ (212 cm^−1^) are ascribed to the external modes (rotation or translation) of BiVO_4_ [46,47]. The Raman peaks present at P_16_ (325 cm^−1^) and P_17_ (367 cm^−1^) are assigned to the asymmetric (B_g_ symmetry) and symmetric (A_g_ symmetry) bending modes of the VO_4_ tetrahedron, respectively [48]. The Raman peaks P_18_ (710 cm^−1^) and P_19_ (826 cm^−1^) are attributed to the asymmetric and symmetric (V-O) stretching modes of BiVO_4_, respectively [[46], [47], [48]]. These characteristics Raman features related to Nb_6_ and BiVO_4_ present in the Nb_6_-BiVO_4_ hybrid indicate successful formation of the Nb_6_-BiVO_4_ hybrid with intactness of Nb_6_ during thin film hybridization.

### 4.2 XPS

**Fig. 3:**
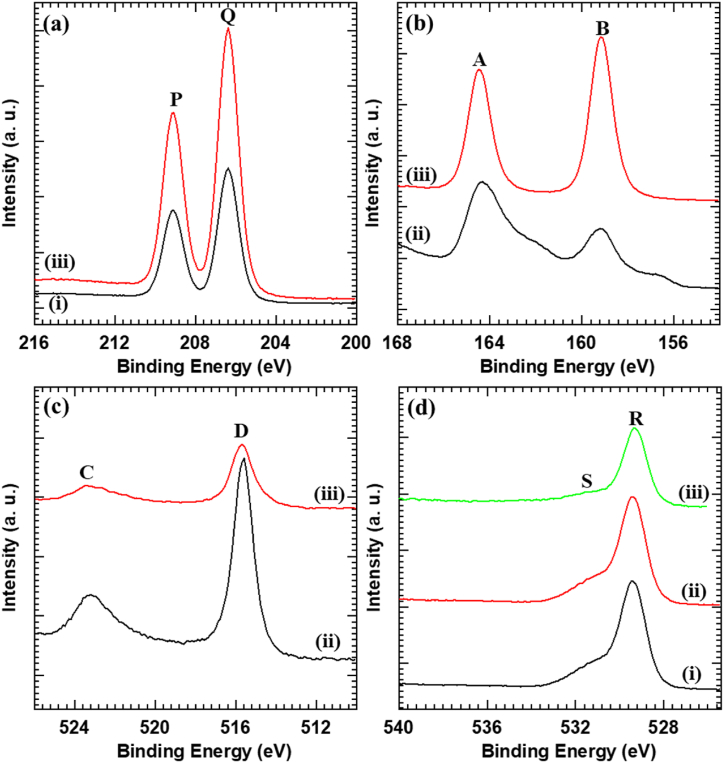
(a) Nb 3d, **(b)** Bi 4f, **(c)** V 2p, and **(d)** O 1s core-level XPS spectra of (i) Nb_6_, (ii) BiVO_4_, and (iii) C-BN2 hybrid thin films.

The effect of the hybridization of Nb_6_ and BiVO_4_ on the chemical bonding characteristics of present Nb_6_-BiVO_4_ hybrids is inspected with XPS analysis. The survey XPS spectrum of C-BN2 thin film (Fig. S2 in ESI) shows the spectral features of the Nb, Bi, V and O elements, indicating the presence of stated elements in C-BN2 thin film. As plotted in Fig. 3(a), the core level Nb 3d XPS spectra of pristine Nb_6_ and Nb_6_-BiVO_4_ hybrid display two sharp peaks at P (207.1 eV) and Q (209.7 eV) correspond to the spin-orbit splitting of Nb 3d_5/2_ and Nb 3d_3/2_, respectively [36]. The position of peaks and their binding energy (BE) difference indicate that Nb in its pentavalent state is present in the above samples [49]. Fig. 3(b) shows the Bi 4f core-level XPS spectra of the pristine BiVO_4_ and Nb_6_-BiVO_4_ hybrid. The spectra display A (164 eV) and B (157.5 eV) peaks corresponding to Bi 4f_5/2_ and Bi 4f_7/2_ states, respectively, which confirms the presence of Bi^3+^ ions in pristine BiVO_4_ and Nb_6_-BiVO_4_ hybrid samples [50]. As shown in Fig. 3(c), the V 2p core-level XPS spectra of BiVO_4_ and Nb_6_-BiVO_4_ hybrid show spectral features C (523.1 eV) and D (515.5 eV) corresponding to the spin-orbit coupling of V 2p_1/2_ and V 2p_3/2_, respectively (BE difference of 7.28 eV), which confirms the presence of pentavalent state of V in pristine BiVO_4_ and Nb_6_-BiVO_4_ hybrid samples [51]. The high-resolution O 1s XPS spectra of pristine Nb_6_, BiVO_4_ and Nb_6_-BiVO_4_ hybrid (Fig. 3(d)) exhibit an intense peak at R (529.5 eV) which is attributed to the presence of oxygen in pristine Nb_6_, BiVO_4_ and Nb_6_-BiVO_4_ hybrid thin films [50]. Moreover, the minor signature at S (532.7 eV) corresponds to the hydroxyl groups arising from the structural or adsorbed water molecules [50]. The deconvoluted XPS spectra of pristine Nb_6_, BiVO_4_ and C-BN2 hybrid thin films are shown in Fig. S3-S6 (ESI). The observed XPS features underscore strong evidence of the presence of Bi^3+^, V^5+^ and Nb^5+^ states of Bi, V and Nb, respectively, which confirms the formation of Nb_6_-BiVO_4_ hybrid thin film. When an electron is transferred from one material to another in a hybrid system, the BE of core-level electrons in the donor or acceptor materials is shifted. In this case, electrons are transferred from BiVO_4_ to Nb_6_; thus, the BEs of core-level electrons in Nb_6_ are slightly changed. This results in a slight shift of the Nb 3d core-level XPS spectra of the C-BN2 hybrid towards lower BEs, suggesting an increase in electron density on Nb_6_ [52]. The intimate electronic coupling between Nb_6_ and BiVO_4_ leads to the improved electron density on Nb_6_.

### 4.3 FESEM and EDS mapping

**Fig. 4:**
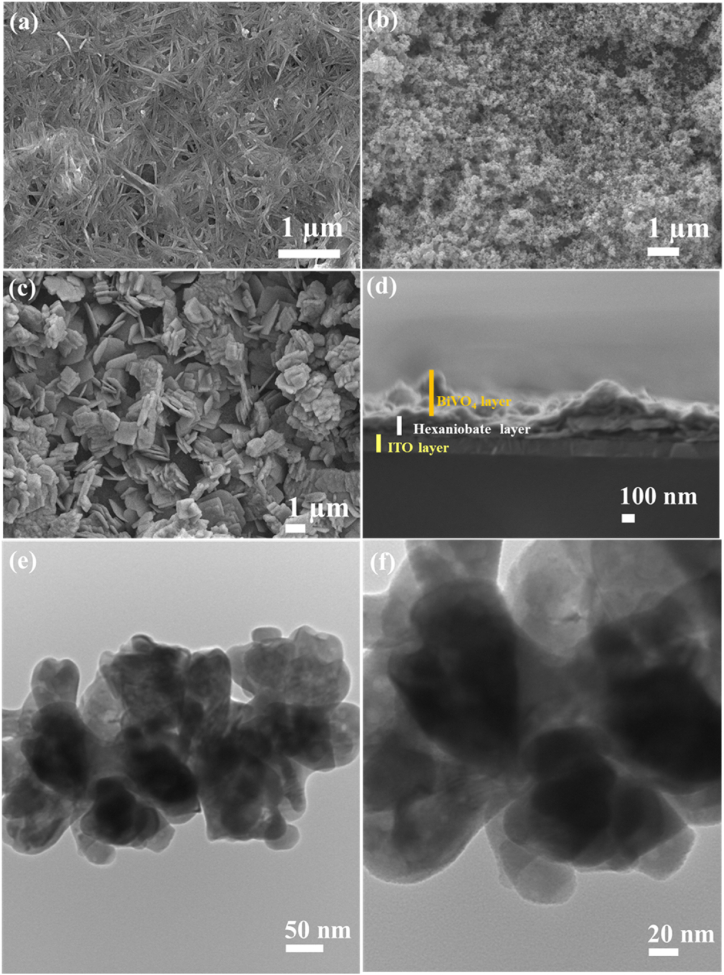
Top view FESEM micrographs of **(a)** Nb_6_, **(b)** BiVO_4_, **(c)** C-BN2 nanohybrid thin films, **(d)** cross-sectional FESEM micrograph of C-BN2 nanohybrid thin film, and **(e**–**f)** HRTEM images of C-BN2 nanohybrid thin film.

The surface morphology, shape and size of present Nb_6_-BiVO_4_ hybrids, as compared to pristine Nb_6_ and BiVO_4_ were studied with FESEM analysis, as shown in Fig. 4. The EPD deposited Nb_6_ thin film (Fig. 4(a)) displays the hexaniobate nanoscrolls laying parallel to the surface of ITO substrate. The lateral dimensions of the nanoscrolls are between 600 and 700 nm. The nanoscrolls are randomly aggregated on the surface of the ITO substrate to form a porous structure of Nb_6_ thin film. The CSG-deposited BiVO_4_ thin film (Fig. 4(b)) shows interconnected nanoparticle morphology with an average particle size within the 75–95 nm range [29]. Conversely, the Nb_6_-BiVO_4_ hybrid (Fig. 4(c)) exhibits highly porous nanoclusters composed of randomly aggregated nanosheets, creating the slit-shaped porous. The edge-to-face interaction of deposited nanosheets forms the highly porous aggregated nanosheet network, creating the house-of-cards type morphology. A close examination of the present FESEM discloses that the average lateral size of the Nb_6_-BiVO_4_ hybrid ranges between 650 and 750 nm. Such a highly porous structure can enable exceptional electron-hole pair separation and transport to catalytic sites, which is beneficial for solar-driven photocatalysis applications. This type of porous morphology is usually reported for 2D nanosheet-based hybrids, which is crucial for solar-driven photo-functional applications [[53], [54], [55], [56]].

Moreover, the chemical composition and the distribution of constituent elements of the Nb_6_-BiVO_4_ hybrid are probed with EDS and elemental mapping analysis, as shown in Fig. S7 and Fig. S8 (ESI). The Nb_6_-BiVO_4_ hybrid thin film shows the uniform distribution of niobium (Nb), bismuth (Bi), vanadium (V) and oxygen (O) elements at the nanometer scale, confirming the uniform growth of BiVO_4_ on the surface of Nb_6_ thin film without any special phase separation. Due to the large thickness of CSG deposited BiVO_4_ thin film, the Nb composition is appeared lower in the C-BN2 hybrid thin film. However, the upper layer BiVO_4_ displays uniform deposition of stoichiometric BiVO_4_ with Bi/V ratio of 0.97.

The morphological features of optimized C-BN2 hybrid thin film are further investigated with HRTEM analysis, as shown in Fig. 4(e and f). The C-BN2 hybrid thin film displays highly porous nanoclusters composed of randomly aggregated nanosheets, creating a house-of-cards type structure matching with FESEM analysis. The distribution of constituent elements of the Nb_6_-BiVO_4_ hybrid thin film is further examined with elemental mapping analysis, as represented in Fig. S9 (ESI). The Nb_6_-BiVO_4_ hybrid thin film displays the uniform distribution of Nb, Bi, V and O elements over the entire mapping region at the nanometer scale, highlighting the uniform growth of BiVO_4_ on Nb_6_ thin film surface without any special phase separation.

### 4.4 UV–vis DRS and photoluminescence spectroscopy

**Fig. 5:**
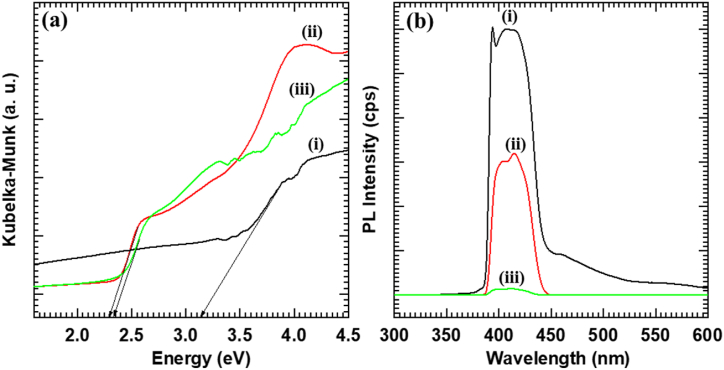
(a) UV–vis DRS, and **(b)** PL spectra of (i) Nb_6_, (ii) BiVO_4_, (iii) C-BN2 nanohybrid thin films.

The electronic structure and optical properties of Nb_6_-BiVO_4_ hybrids and pristine Nb_6_ and BiVO_4_ are examined using UV–vis DRS analysis. As represented in Fig. S10 (ESI) and Fig. 5(a), the pristine BiVO_4_ thin film shows strong visible light absorption with band gap energy of 2.33 eV, which can be ascribed to the transition of electrons from hybrid Bi 6s-O 2p orbital to V 3d orbital of BiVO_4_ [57]. In contrast, the pristine Nb_6_ thin film displays prominent absorption in the UV region, which differs from the optical profile of BiVO_4_. On the other hand, the Nb_6_-BiVO_4_ hybrid thin film shows a quite similar type of optical behavior as that of pristine BiVO_4_ thin film. Compared with the pristine BiVO_4_, the Nb_6_-BiVO_4_ hybrid causes a more prominent absorption of visible light with a band gap energy of 2.29 eV, highlighting its superior optical characteristics. This observation demonstrates that the Nb_6_-BiVO_4_ hybrids can be highly effective photocatalysts for visible-light-driven photocatalytic dye degradation. It is concluded that the improved visible light harvesting nature of present hybrids can be attributed to the spatial electronic coupling between Nb_6_ and BiVO_4_ [[53], [54], [55]].

The electron transfer between Nb_6_ and BiVO_4_ is examined with a change in PL signal upon hybridization [53,54]. As shown in Fig. 5(b), the PL intensity of BiVO_4_ is significantly quenched after hybridization with Nb_6_, clearly highlighting depressed electron-hole recombination. In the present hybridization strategy, electron-hole recombination is decreased due to the effective separation of photoinduced electrons and holes. The intimate electronic coupling between BiVO_4_ nanoparticles and hexaniobate nanoscrolls resulted in the enhanced photocatalytic activity of Nb_6_-BiVO_4_ hybrid thin films.

### 4.5 Photocatalytic dye degradation performance

**Fig. 6:**
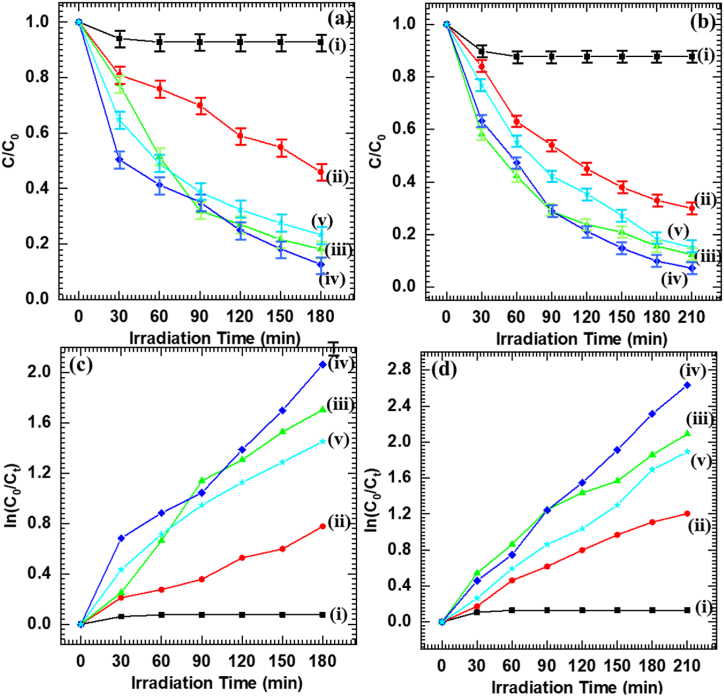
Photocatalytic degradation performance of **(a)** MB, **(b)** Rh-B, and Pseudo-first-order kinetics of **(c)** MB, **(d)** Rh-B for (i) Nb_6_, (ii) BiVO_4_, (iii) C-BN1, (iv) C-BN2, (v) C-BN3 hybrid thin-film photocatalysts (Error bar, mean ± standard deviation, n = 3).

The photocatalytic activity of Nb_6_-BiVO_4_ hybrids is examined with photocatalytic degradation of MB and Rh-B dyes in the presence of visible light illumination. The photocatalytic dye degradation performance of pristine Nb_6_ and BiVO_4_ thin films is also measured to analyze the effect of hybridization on photocatalytic activity. The UV–vis absorption spectra and photocatalytic degradation performance of pristine Nb_6_, BiVO_4_ and Nb_6_-BiVO_4_ hybrid thin films are shown in Fig. S11-S12 (ESI) and Fig. 6(a and b) [58]. The pristine Nb_6_ thin film shows minor photocatalytic degradation in visible light due to its wide band gap energy. On the contrary, the pristine BiVO_4_ thin film exhibited superior photocatalytic degradation of MB and Rh-B dyes compared to Nb_6_ thin film. The photocatalytic activity of pristine BiVO_4_ is further enhanced after hybridization with Nb_6_. The optimized C-BN2 hybrid thin film showed excellent photocatalytic degradation activity for both MB and Rh-B dyes with photodegradation rates of 87.3 and 92.8 %, respectively, which are superior to that of pristine BiVO_4_ (54 and 70 %). Although the pristine BiVO_4_ is a highly active photocatalyst for visible-light-induced photocatalytic degradation of recalcitrant molecules, the present Nb_6_-BiVO_4_ hybrids with exceptional photocatalytic activity can be considered as highly efficient visible-light-induced photocatalysts for photodegradation of various of organic molecules.

The enhanced photocatalytic degradation performance of BiVO_4_ after hybridization with highly stable Nb_6_ can be linked with the intimate electronic coupling between Nb_6_ and BiVO_4_, which facilitates enhanced electron density, advantageous for effective photocatalytic reactions. The expanded surface area obtained after hybridization by forming a highly porous house-of-cards type morphology can promote efficient electron-hole transport and plenty of reaction sites, which boost photocatalytic activity. Conclusionally, the superior photocatalytic activity of present hybrid thin films can be attributed to the effective electronic coupling with easy charge transport, high photostability, strong visible light harvesting ability, expanded surface area and highly porous house-of-cards type structure useful for visible-light-driven photocatalytic dye degradation.

The photodegradation performance of MB and Rh-B was further described by their kinetics using a pseudo-first-order rate kinetic model [59,60]. Fig. 6(c and d) represents the pseudo-first-order reaction kinetics for MB and Rh-B dyes. The k and linear correlation coefficient (R^2^) values of all tested thin-film photocatalysts for MB and Rh-B degradation are noted in Table S2 (ESI). Compared with other photocatalysts, the C-BN2 hybrid thin film displays an improved k value, signifying the maximum photocatalytic activity. The highest k values obtained for the C-BN2 hybrid thin film for MB and Rh-B degradation are 0.0115 and 0.0146 min^−1^, respectively.

### 4.6 Recyclability study for MB and Rh-B

**Fig. 7:**
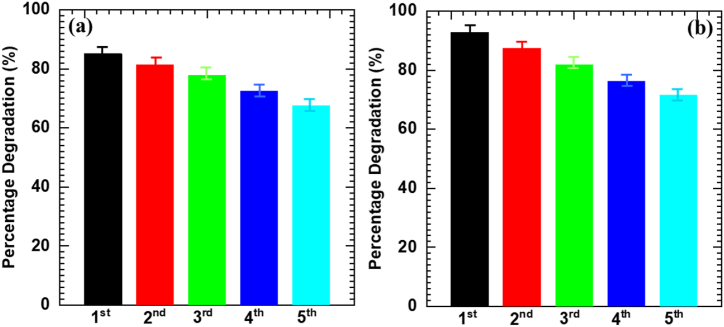
Recyclability of C-BN2 hybrid thin film for **(a)** MB, and **(b)** Rh-B dyes (Error bar, mean ± standard deviation, n = 3).

The photostability of C-BN2 hybrid thin film for photocatalytic degradation of MB and Rh-B dyes is estimated with the course of five consecutive degradation cycles as displayed in Fig. 7. The present C-BN2 hybrid thin film shows superior recycling performance for MB and Rh-B photodegradation with degradation rates of 66.8 and 71.5 % within 180 and 210 min of visible light exposure, respectively. The C-BN2 hybrid thin film retains its photocatalytic activity with a negligible decrease for five repetitive degradation cycles, suggesting its superior photostability. The negligible decrement in degradation performance after five consecutive cycles is attributed to the adsorption of dye molecules on the surface of the C-BN2 hybrid thin film and the detachment of loosely bound photocatalyst particles from the film surface.

The above results validate the superior photocatalytic activity of C-BN2 hybrid thin film for the photodegradation of MB and Rh-B dyes under visible light illumination. Furthermore, its photocatalytic activity is discovered for the degradation of antibiotic.

### 4.7 Photocatalytic antibiotic degradation performance

**Fig. 8:**
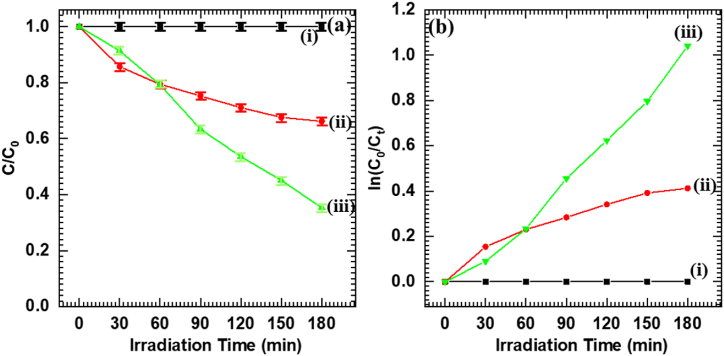
(a) Degradation performance, and **(b)** Pseudo-first-order kinetics of TC over (i) Nb_6_, (ii) BiVO_4_ and (iii) C-BN2 hybrid thin film (Error bar, mean ± standard deviation, n = 3).

The photocatalytic activities of the pristine Nb_6_, BiVO_4_ and optimized C-BN2 hybrid thin films were further evaluated with photodegradation of TC as the target antibiotic for decomposition [52]. Fig. S13 (ESI) shows their respective time-dependent UV–vis absorption spectra. The photodegradation efficiency of the above photocatalysts for TC degradation is calculated using equation (1). Fig. 8(a) shows that C-BN2 hybrid thin film exhibits improved photocatalytic degradation performance for TC (64.7 %) as compared to pristine BiVO_4_ (33.8 %) under 180 min of visible light exposure. Fig. 8(b) displays the pseudo-first-order reaction kinetics of TC. The k and R^2^ values of the tested samples for TC degradation are given in Table S3 (ESI). The C-BN2 hybrid thin film shows the highest k and R^2^ values of 0.0058 min^−1^ and 0.9942, respectively. The present results strongly evidence the usefulness of Nb_6_-BiVO_4_ hybrid thin films for antibiotic degradation.

### 4.8 Charge trapping experiments

**Fig. 9:**
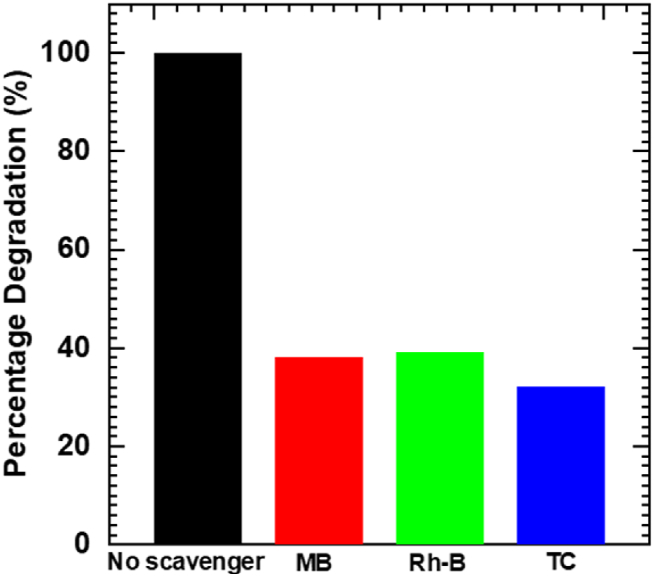
Hydroxyl radicals trapping experiment results compared to the reaction in absence of isopropanol scavenger for MB, Rh-B and TC degradation.

The substantial improvement in the photocatalytic activity of Nb_6_-BiVO_4_ hybrids is attributed to the intimate electronic coupling between Nb_6_ and BiVO_4_. Thus, charge-trapping experiments were conducted to identify the active species responsible for MB, Rh-B dyes and TC antibiotic degradation over the Nb_6_-BiVO_4_ hybrid catalyst. These scavenger tests were conducted using suitable scavengers under similar experimental conditions used for photocatalytic experiments. The hydroxyl radicals trapping experiments were conducted to identify the presence of hydroxyl radicals in MB, Rh-B and TC degradation over Nb_6_-BiVO_4_ hybrid thin film catalyst. Isopropanol (C_3_H_8_O) (concentration: 1 mmol L^−1^) was used as a scavenger for hydroxyl radicals (OH^−^) radicals [14,61,62]. Fig. 9 displays a scavenging test graph representing relative % degradation compared to the reaction in the absence of a scavenger for photodegradation of MB, Rh-B dyes and TC antibiotic. It is observed that a significant decrease in photocatalytic activity of the Nb_6_-BiVO_4_ hybrid is obtained upon the addition of isopropanol scavenger. It indicates that OH^−^ radicals are the main reactive species for the photocatalytic degradation of MB, Rh-B dyes and TC antibiotic.

### 4.9 Mineralization study using COD analysis

The COD analysis allows the measurement of waste in terms of the total quantity of oxygen required for the oxidation of organic matter to CO_2_ and water. The COD of the dyes (MB, Rh-B) and antibiotic (TC) solutions was estimated before and after the degradation experiments. The percentage removal of COD of the MB, Rh-B dyes and TC antibiotic over optimized Nb_6_-BiVO_4_ hybrid thin film was estimated using equation (3). It is observed that the COD values decreased from 1920 to 320 mg L^−1^, 960 to 140 mg L^−1^, and 880 to 340 mg L^−1^ for MB, Rh-B and TC, respectively. The percentage removal of COD is 83.33, 85.42, and 61.36 % for MB, Rh-B dyes and TC antibiotic, respectively. The reduction in the COD values of the degraded dye and antibiotic solutions shows the mineralization of MB, Rh-B and TC molecules along with color removal. The present results highlight that both degradation and mineralization play significant roles in photocatalytic activity under visible light irradiation.

### 4.10 Photocatalytic degradation mechanism

**Fig. 10:**
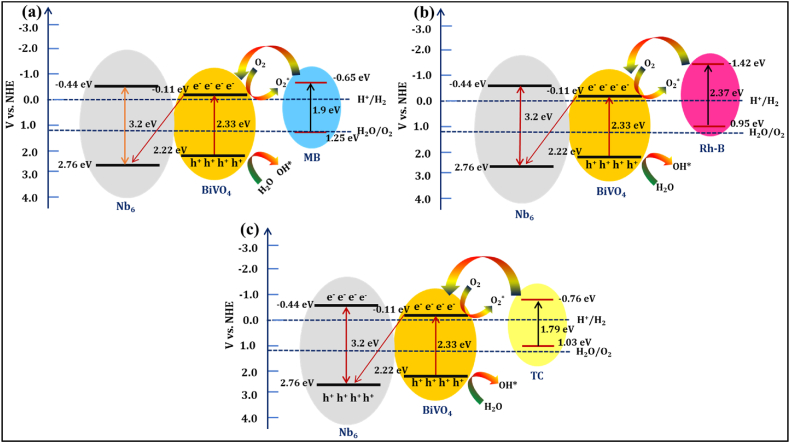
Schematic representation of photocatalytic multitargeted degradation mechanism of **(a)** MB, **(b)** Rh-B dyes and **(c)** TC antibiotic over Nb_6_-BiVO_4_ hybrids.

The electron transfer between Nb_6_ and BiVO_4_ in the Nb_6_-BiVO_4_ hybrids is investigated with band structure estimated from the cyclic voltammetry (CV) measurements and UV–vis DRS [29,63,64]. The conduction band (CB) and valence band (VB) positions of Nb_6_ and BiVO_4_ were calculated using equations S1-S3 explained in **Note S1** (ESI) [14,61]. The photocatalytic multitargeted degradation mechanism is mainly based on the photoinduced charge separation and transport to the semiconductor surface upon exposure to visible light irradiations [65]. When photons with energy equal to or greater than the band gap of BiVO_4_ are incident on the Nb_6_-BiVO_4_ hybrids, electrons from the VB of BiVO_4_ are excited to the CB and electron-hole pairs are generated. It is observed that the Nb_6_ shows a higher position for CB and VB as compared to CB and VB of BiVO_4_. The electron transfer between Nb_6_ and BiVO_4_ in the Nb_6_-BiVO_4_ hybrids is based on the Z-scheme heterostructure. The photogenerated electrons from the CB of BiVO_4_ can transferred into the VB of Nb_6_, resulting in the effective electron-hole pair separation with depressed electron-hole recombination. The resultant decreased electron-hole recombination is evidenced by the highly quenched PL signal upon hybridization. The holes at the VB of BiVO_4_ generate hydroxyl radicals (OH∗) when reacting with H_2_O molecules. Generated OH∗ radicals are strong oxidizing agents that can degrade the adsorbed dye (MB and Rh-B) and antibiotic (TC) molecules. On the other hand, superoxide radicals (∗O_2_^−^) are produced from the reaction of O_2_ with photogenerated electrons from CB of BiVO_4_. Again, hydroperoxyl radicals (HOO∗) are generated from the protonation of superoxide radicals. Finally, H_2_O_2_ generated from hydroperoxyl radicals dissociates into OH∗ radicals, which can degrade the target dye and antibiotic molecules [66]. The schematic representation of the photocatalytic multitargeted degradation mechanism over Nb_6_-BiVO_4_ hybrids is shown in Fig. 10.

(3b) $${Nb}_{6}-BiVO_{4}+h\;\nu \to \;e^{-}+h^{+}$$

(4) $$h^{+}+OH^{-}/H_{2}O\;\to \;OH^{*}\;/\;OH^{*}+{\;H}^{+}$$

(5) $$O_{2}+e^{-}\;\to *{O_{2}}^{-}$$

(6) $$*{O_{2}}^{-}+H^{+}\;\to \;HOO*$$

(7) $$2HOO*\;\to \;H_{2}O_{2}+O_{2}$$

(8) $$H_{2}O_{2}\;\to \;{2OH}^{*}$$

(9) $$\text{Organic}\;\text{molecule}+\text{OH}*\to \;{\text{CO}}_{2}+H_{2}O$$

## 5 Conclusions

The CSG of BiVO_4_ layers over EPD-deposited Nb_6_ thin films yields highly porous Nb_6_-BiVO_4_ hybrid thin films with superior photoactivity for visible-light-driven photocatalytic degradation of MB, Rh-B dyes and TC antibiotic. The resultant hybrid thin films show strong visible light harvesting ability, effective electronic coupling between Nb_6_ and BiVO_4_ with highly quenched PL signal, high photostability, and highly porous house-of-cards type morphology beneficial for visible-light-induced photocatalytic activity. Consequently, the best optimized Nb_6_-BiVO_4_ hybrid thin film photocatalyst displays excellent photocatalytic degradation activity for MB, Rh-B dyes and TC antibiotic with photodegradation rates of 87.3, 92.8 and 64.7 %, respectively, which are higher than that of pristine BiVO_4_ thin film photocatalyst (54, 70 and 33.8 %). In the present hybridization strategy, electron-hole recombination is lowered owing to the effective separation of photoinduced electrons and holes caused by the intimate electronic coupling between Nb_6_ and BiVO_4_. The creation of a high surface area mesoporous house-of-cards type structure can provide plenty of reaction sites and easy electron-hole transport, resulting in improved photocatalytic activity. The present findings vividly highlight the usefulness of thin film hybridization for improving the photocatalytic properties of pristine BiVO_4_ by minimizing its charge recombination rate and enhancing photostability.

## CRediT authorship contribution statement

**Shirin P. Kulkarni:** Investigation, Methodology, Writing-original manuscript. **Vikas V. Magdum:** Data curation. **Yogesh M. Chitare:** Validation. **Dhanaji B. Malavekar:** Resources. **Jin H. Kim:** Resources. **Sultan Alshehri:** Resources, Funding acquisition. **Jayavant L. Gunjakar:** Conceptualization, Supervision, Funding acquisition, Writing – review & editing. **Shashikant P. Patole:** Funding acquisition, Writing – review & editing.

## Declaration of competing interest

The authors declare that they have no known competing financial interests or personal relationships that could have appeared to influence the work reported in this paper.
