# Supplementary material for: 2D porous hexaniobate-bismuth vanadate hybrid photocatalyst for photodegradation of aquatic refractory pollutants
Source: Heliyon. 2024 Oct 10;10(20):e39235. doi: 10.1016/j.heliyon.2024.e39235 (PMC11532252; doi:10.1016/j.heliyon.2024.e39235)
Supplement: Multimedia component 1 [file mmc1.pdf]

2D Porous Hexaniobate-Bismuth Vanadate Hybrid Photocatalyst for Photodegradation of Aquatic Refractory Pollutants

Shirin P. Kulkarni[a], Vikas V. Magdum[a], Yogesh M. Chitare[a], Dhanaji B. Malavekar[b], Jin H. Kim[b], Sultan Alshehri[c], Jayavant L. Gunjakar[a,*], and Shashikant P. Patole[d,*]

[a]*Centre for Interdisciplinary Research, D. Y. Patil Education Society (Deemed to be University), Kolhapur - 416 006, MS, India*

[b]*Optoelectronic Convergence Research Centre, Department of Materials Science and Engineering, Chonnam National University, Gwangju, 61186, South Korea*

[c]*Department of Pharmaceuticals, College of Pharmacy, King Saud University, Riyadh 11451, Saudi Arabia*

[d]*Department of Physics, Khalifa University of Science and Technology, Abu Dhabi 127788, United Arab Emirates*

[*]Corresponding Authors:

Email: jlgunjakar@gmail.com (Jayavant L. Gunjakar); shashikant.patole@ku.ac.ae (Shashikant P. Patole)

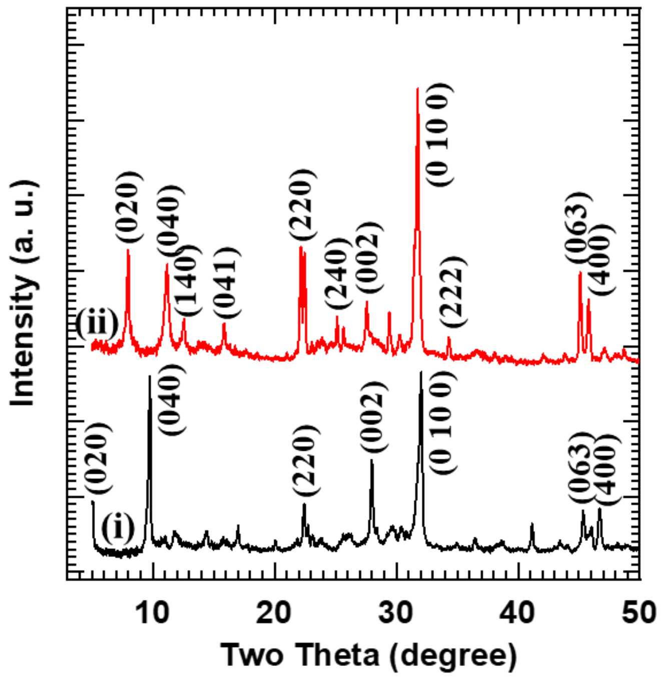

Fig. S1: XRD patterns of (i) potassium hexaniobate and (ii) protonated hexaniobate.

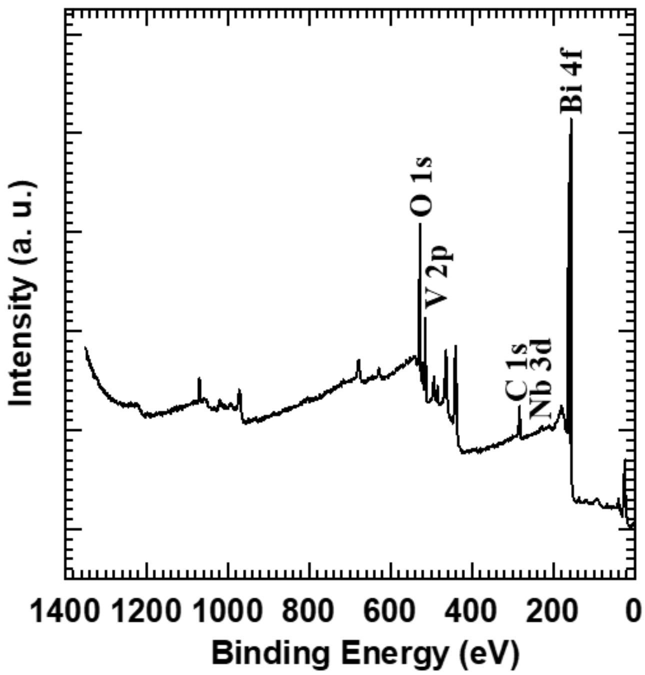

Fig. S2: Survey XPS spectrum of C-BN2 nanohybrid thin film.

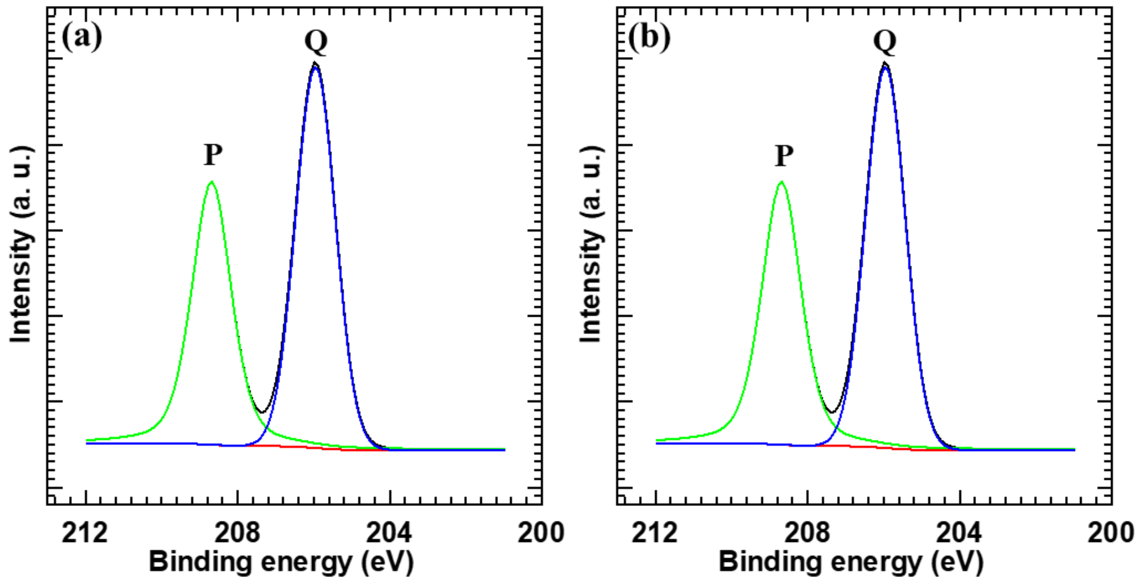

Fig. S3: Deconvoluted Nb 3d core level XPS spectra of **(a)** Nb_6 and **(b)** C-BN2 nanohybrid thin films.

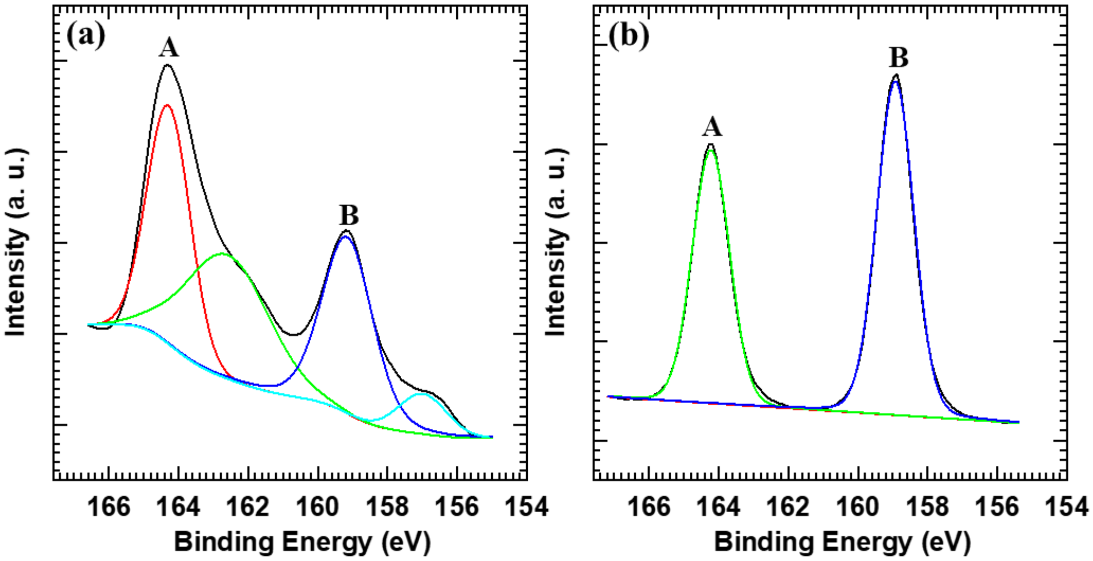

Fig. S4: Deconvoluted Bi 4f core level XPS spectra of **(a)** $BiVO_4$, and **(b)** C-BN2 nanohybrid thin films.

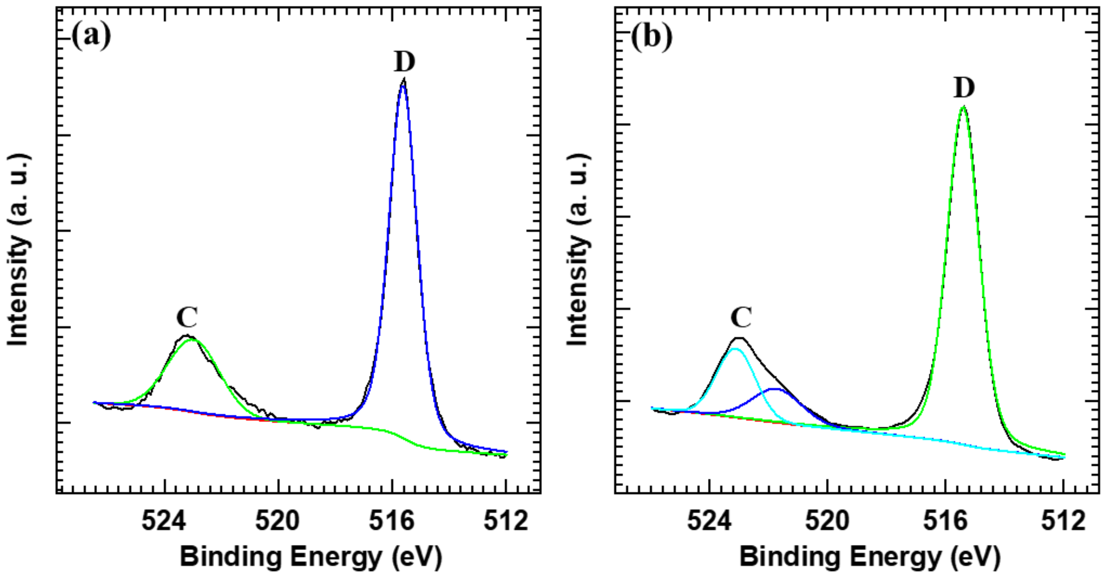

Fig. S5: Deconvoluted V 2p core level XPS spectra of **(a)** $BiVO_4$, and (b) C-BN2 nanohybrid thin films.

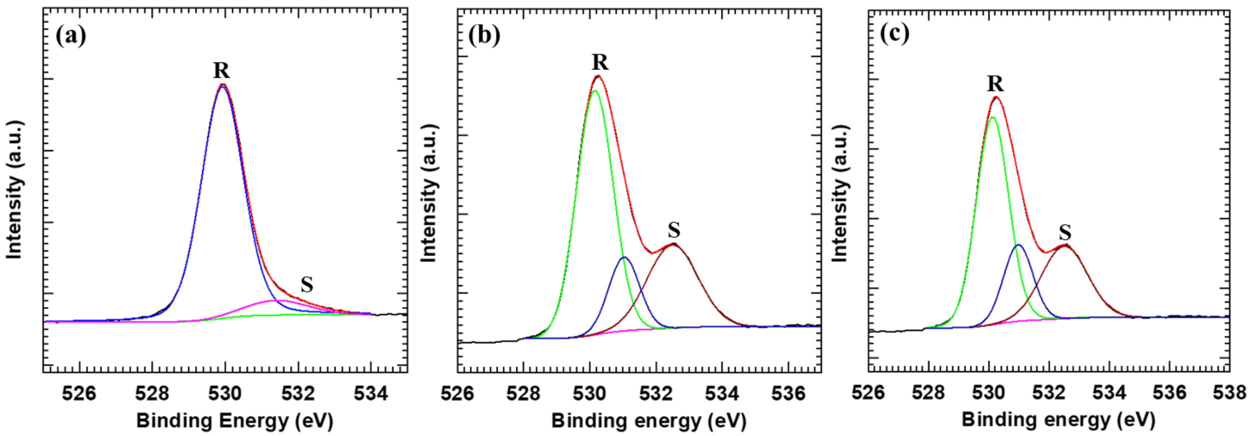

Fig. S6: Deconvoluted O 1s core level XPS spectra of **(a)** Nb_6, **(b)** $BiVO_4$, and **(c)** C-BN2 hybrid thin films.

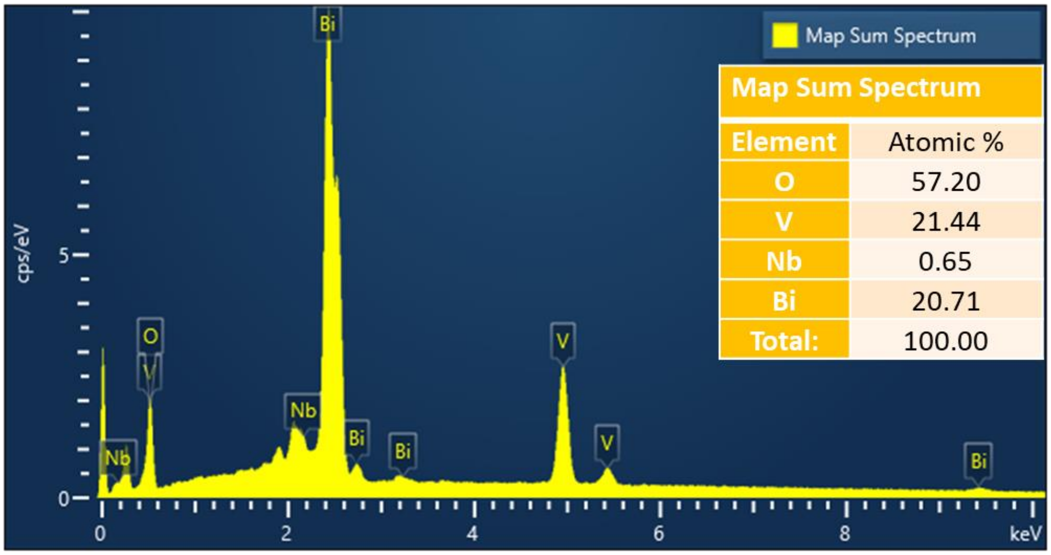

Fig. S7: EDS spectrum of C-BN2 hybrid thin film.

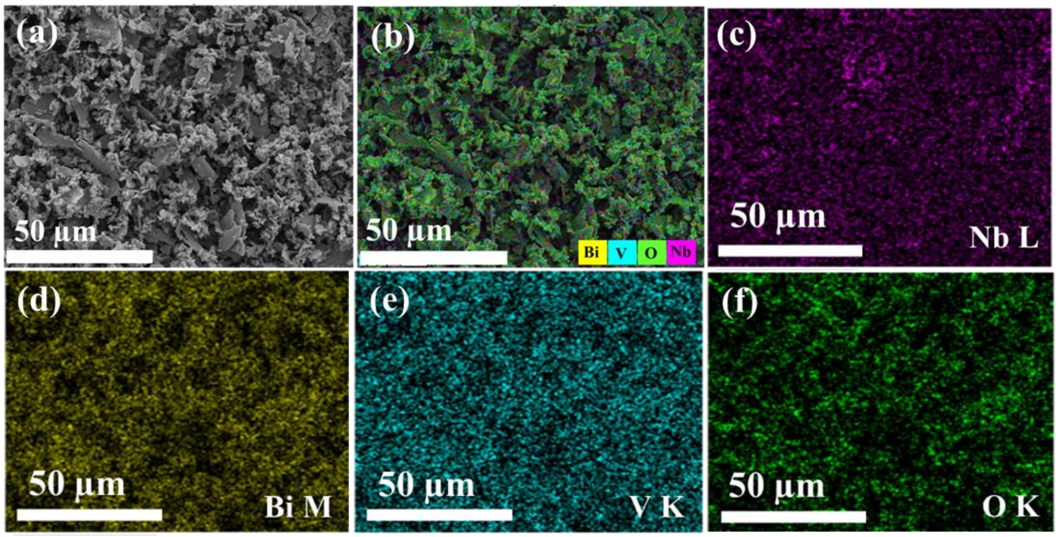

Fig. S8: (a) FESEM image, and **(b-f)** EDS elemental maps of C-BN2 nanohybrid thin film.

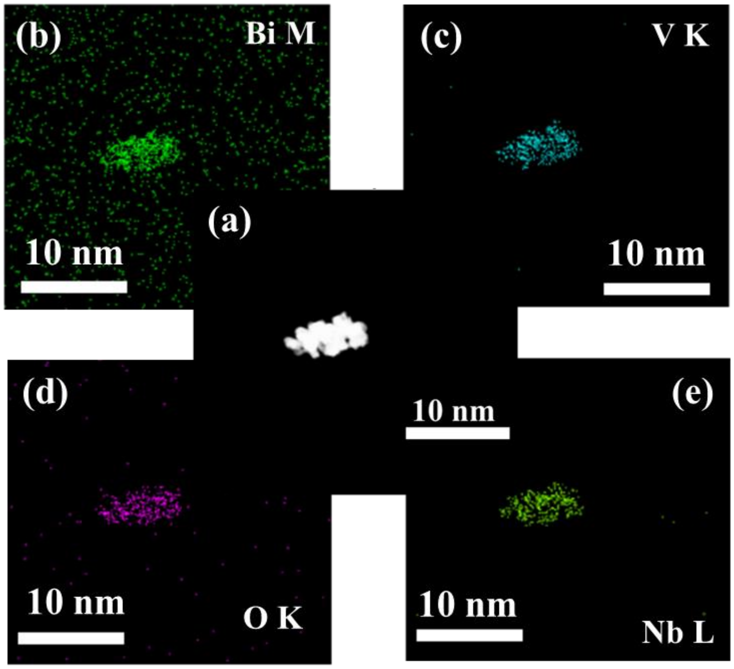

Fig. S9: (a) HRTEM image and (b-e) Elemental maps of C-BN2 nanohybrid thin film.

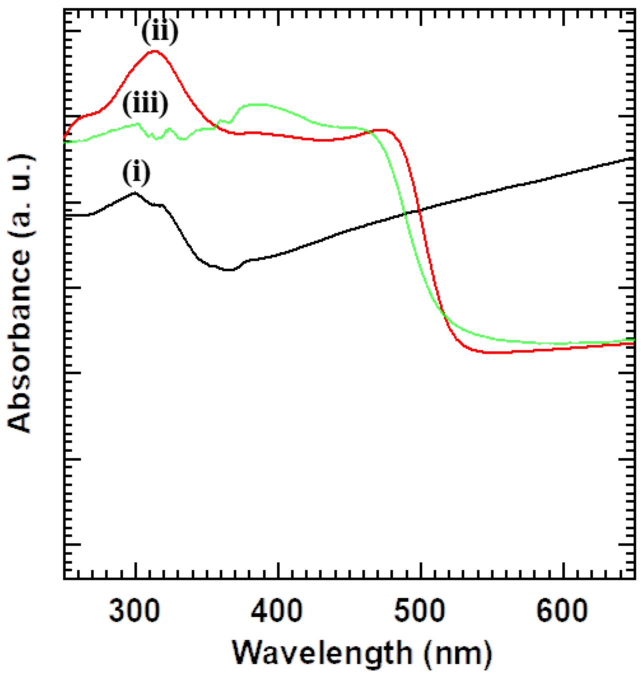

Fig. S10: UV-vis absorbance spectra of (i) Nb_6, (ii) $BiVO_4$, (iii) C-BN2 hybrid thin films.

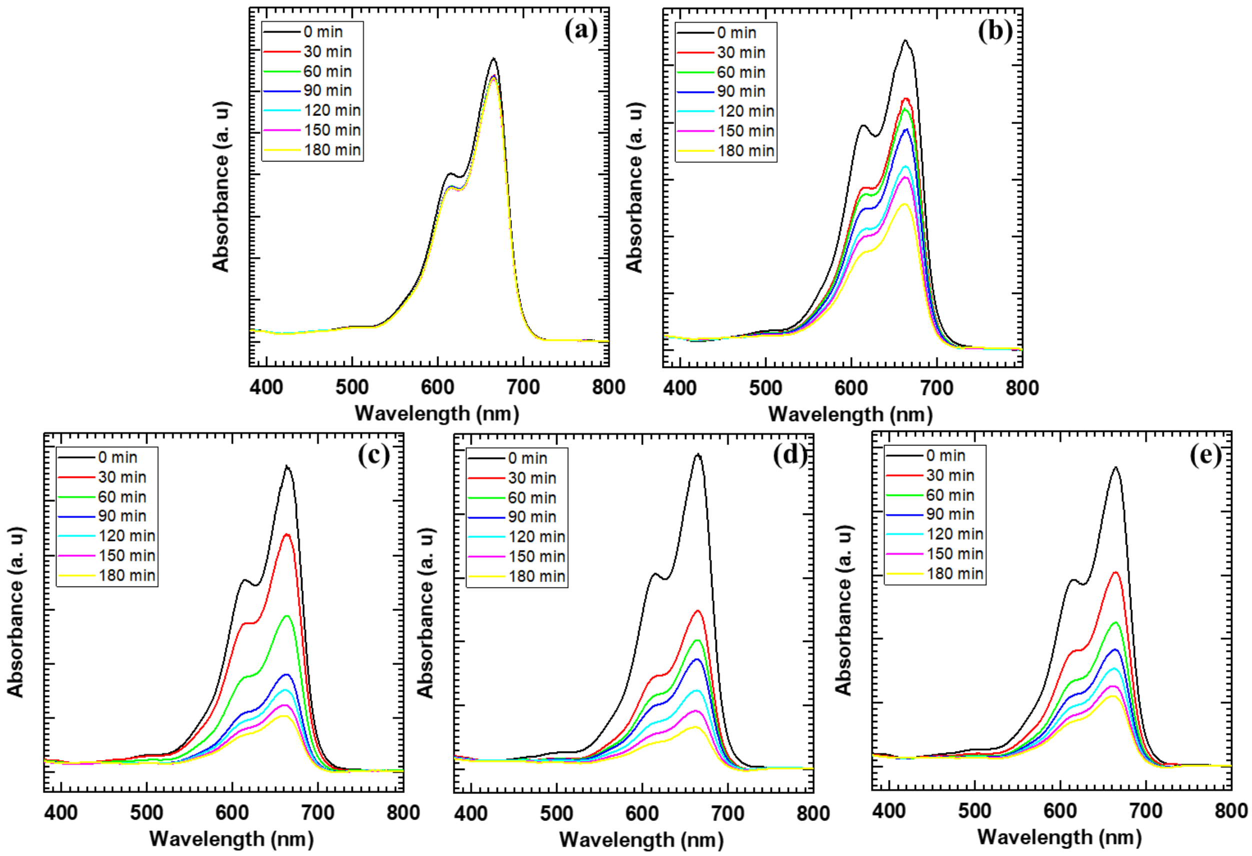

Fig. S11: Time-dependent UV-vis absorption spectra of **(a)** Nb_6, **(b)** $BiVO_4$, **(c)** C-BN1, **(d)** C-BN2, and **(e)** C-BN3 hybrid thin films for MB degradation.

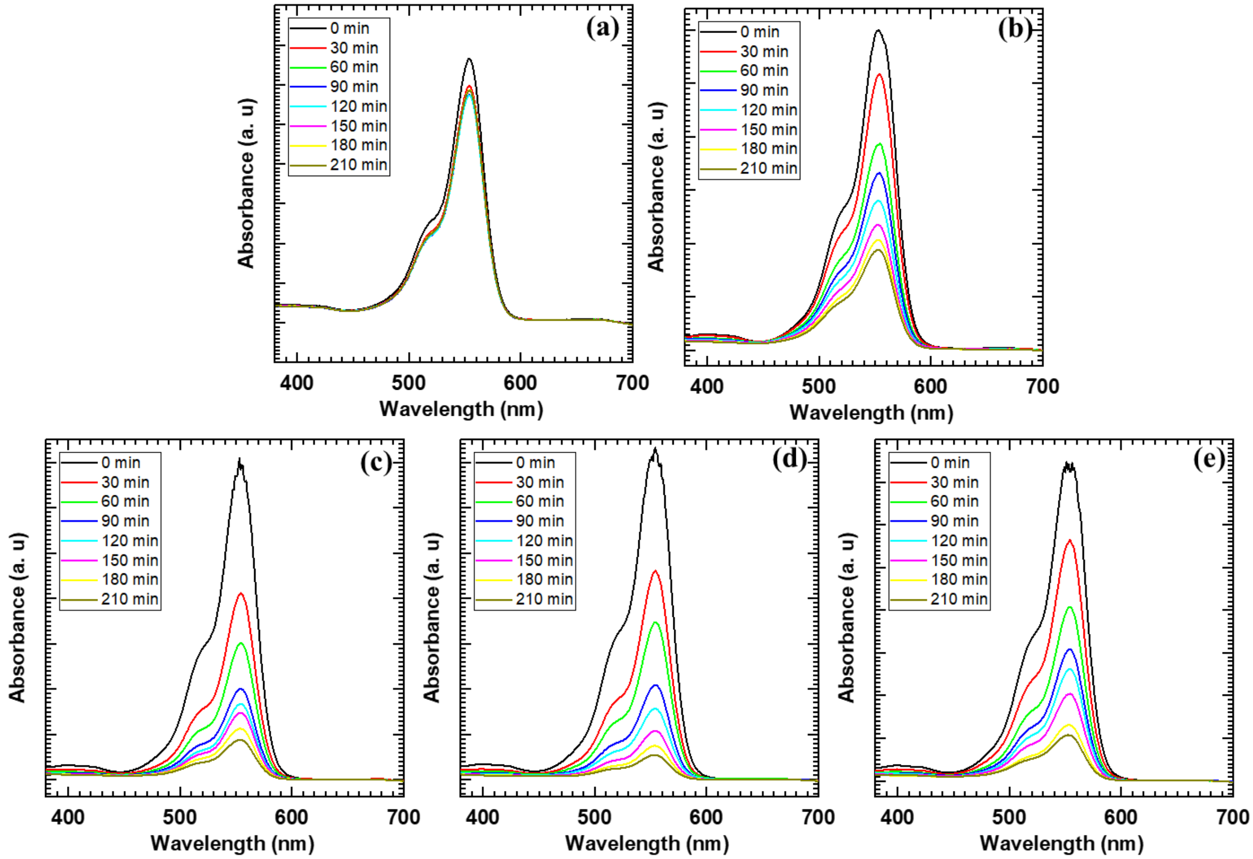

Fig. S12: Time-dependent UV-vis absorption spectra of **(a)** Nb_6, **(b)** $BiVO_4$, **(c)** C-BN1, **(d)** C-BN2, and **(e)** C-BN3 nanohybrid thin films for Rh-B degradation.

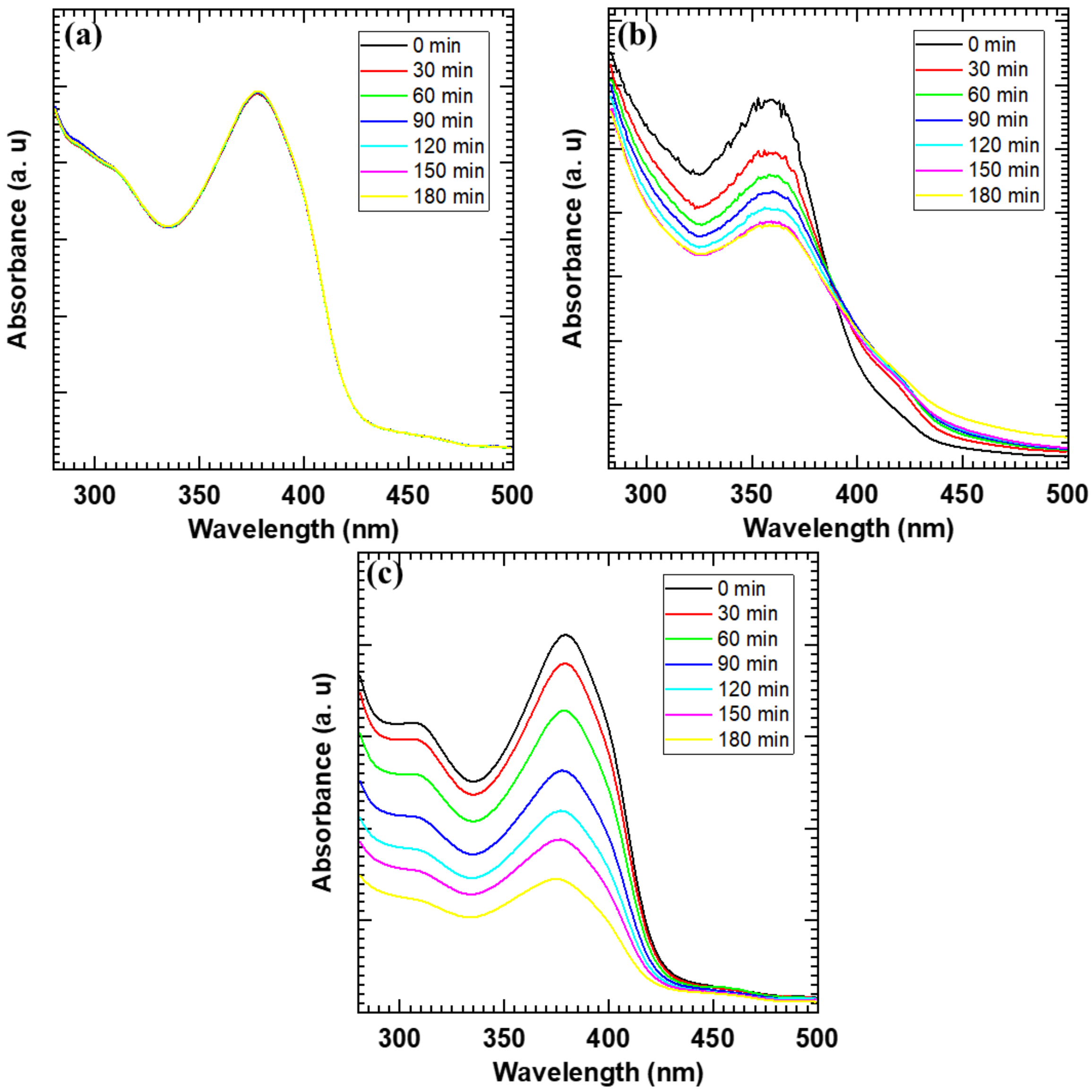

Fig. S13: Time-dependent UV-vis absorption spectra of **(a)** Nb_6, **(b)** $BiVO_4$, and **(c)** C-BN2 nanohybrid thin films for TC degradation.

Table S1: Nb_6 synthesis conditions.

Methods			
1) Solid-state reaction	2) Protonation	3) Intercalation	4) Exfoliation
Molar ratio: K_2CO_3:Nb_2O_5=2:3 Calcination temperature: 1050°C Time: 24 hr	Socking in 1 M HCl for 24 hr (Repeated four-time)	Protonated hexaniobate (0.025 M), TBAOH (1.5 M)	Vigorous shaking for 2 days

Table S2: k and R^2 values of all tested photocatalysts for MB and Rh-B degradation.

Sample	MB		Rh-B	
	**k (min^{-1})**	**R^2**	**k (min^{-1})**	**R^2**
Nb_6	0.0004	0.9996	0.0007	0.9993
$BiVO_4$	0.0043	0.9957	0.0067	0.9933
C-BN1	0.0095	0.9905	0.0116	0.9884
C-BN2	0.0115	0.9885	0.0146	0.9854
C-BN3	0.0081	0.9919	0.0105	0.9895

Table S3: k and R^2 values of tested photocatalysts for TC degradation.

Sample	k (min^{-1})	R^2
Nb_6	0	1
$BiVO_4$	0.0023	0.9977
C-BN2	0.0058	0.9942

Table S4: Comparative study between the present photocatalytic system and previously reported works:

Sr. No.	Photocatalyst	Method	Cata -lyst (mg)	Conc. (Quantity)	Target molecule	Degra-dation (%)	Time (min)	Ref.
1	Nb_3O_7F/Nb_2O_5	Thermal decomposition	20	10 mg L^{-1} (200 ml)	MB	96.7	60	1
2	$AgI/BiVO_4$	Deposition-precipitation	30	20 mg L^{-1} (100 ml)	TC	94.91	60	2
3	$Bi/BiVO_4$-CdS	Chemical bath deposition	20	20 mg L^{-1} (50 ml)	TC	85.5	60	3
4	$BiOI/BiVO_4$	In situ growth	30	5 mg L^{-1} (50 ml)	Rh-B	97	75	4
5	$BiVO_4/BiO_X/Pd$	Single pot synthesis	20	10 mg L^{-1}	Rh-B	100	12	5
6	$BiVO_4@CuO_x$	In situ chemical deposition	-	20 mg L^{-1} (100 ml)	TC	81	20	6
7	$BiVO_4/Fe_3O_4/$ rGO	Hydrothermal	10	10 mg L^{-1} (100 ml)	Rh-B	99	120	7
8	$BiVO_4/InVO_4$	Hydrothermal	30	2 mol L^{-1} (100 ml)	Rh-B	98	40	8
9	$BiVO_4/Zn_2SnO_4$	Hydrothermal	50	10 mg L^{-1} (100 ml)	MB	99.3	80	9
10	CeO_2-Nb_2O_5	Plasma electrolytic oxidation	-	5 mg L^{-1}	MB	58	210	10
11	g-C_3N_4/Nb_2O_5	Hydrothermal	5	10 mg L^{-1} (20 ml)	MB	59	210	11
12	g-C_3N_4/Nb_2O_5	Sonochemical	10	10 mg L^{-1}	Rh-B	81	90	12

				(20 ml)				
13	Nb_2O_5/carbon xerogel	Wet chemical	250	10 mg L^{-1} (500 ml)	MB	68	330	13
14	Nb_2O_5/g-C_3N_4	One-step heating	100	20 mg L^{-1} (100 ml)	TC	90.1	150	14
15	Nb_2O_5/Nb_2CT_x	Hydrothermal	25	10 mg L^{-1} (25 ml)	Rh-B	98.5	120	15
					TC	91.2	180	
16	Nb_6-$BiVO_4$	Chemical solution growth	Thin film	50 µM (3 ml)	MB	87.3	180	This work
				50 µM (3 ml)	Rh B	92.8	210	
				50 µM (3 ml)	TC	64.7	180	

Note S1:

The conduction band (CB) and valence band (VB) potentials of $BiVO_4$ and Nb_6 thin films can be estimated by using the formula,

$$\chi = (A^a + B^b + C^c)^{1/a+b+c} \quad \text{(S1)}$$

$$E_{CB} = \chi - E_e - 0.5E_g \quad \text{(S2)}$$

$$E_{VB} = E_{CB} + E_g \quad \text{(S3)}$$

Where E_{CB}, E_{VB}, E_g, and E_e are the CB potential, VB potential, energy bandgap, and free electron energy on the hydrogen scale (4.5 eV), respectively. χ is the electronegativity of a semiconductor. A, B, and C are the absolute electronegativity of atoms and a, b, and c are the number of atoms in a semiconductor, respectively.

Thus, the calculated CB and VB potentials of $BiVO_4$ thin film are -0.11 and 2.22 eV, respectively. Similarly, the calculated CB and VB potentials of Nb_6 thin film are -0.44 and 2.76 eV, respectively.

References:

[1] Huang, F.; Zhao, H.; Yan, A.; Li, Z.; Liang, H.; Gao, Q.; Qiang, Y. In situ thermal decomposition for preparation of Nb_3O_7F/Nb_2O_5 hybrid nanomaterials with enhanced photocatalytic performance. *J. Alloys Compd.* **2017**, 695, 489-495.

[2] Chen, F.; Yang, Q.; Sun, J.; Yao, F.; Wang, S.; Wang, Y.; Wang, X.; Li, X.; Niu, C.; Wang, D.; Zeng, G. Enhanced photocatalytic degradation of tetracycline by AgI/$BiVO_4$ heterojunction under visible-light irradiation: Mineralization efficiency and mechanism. *ACS Appl. Mater. Interfaces* **2016**, 8, 32887-32900.

[3] Xue, Y.; Chen, Z.; Wu, Z.; Tian, F.; Yu, B. Hierarchical construction of a new Z-scheme Bi/$BiVO_4$-CdS heterojunction for enhanced visible-light photocatalytic degradation of tetracycline hydrochloride. *Sep. Purif. Technol.* **2021**, 275, 119152.

[4] Ni, S.; Zhou, T.; Zhang, H.; Cao, Y.; Yang, P. BiOI/$BiVO_4$ two-dimensional hetero-nanostructures for visible light photocatalytic degradation of rhodamine B. *ACS Appl. Nano Mater.* **2018**, 1, 5128-5141.

[5] Olagunju, M.O.; Zahran, E.M.; Reed, J.M.; Zeynaloo, E.; Shukla, D.; Cohn, J.L.; Surnar, B.; Dhar, S.; Bachas, L.G.; Knecht, M.R. Halide effects in $BiVO_4/BiO_X$ heterostructures decorated with Pd nanoparticles for photocatalytic degradation of rhodamine B as a model organic pollutant. *ACS Appl. Nano Mater.* **2021**, 4, 3262-3272.

[6] Cui, Z.; Wang, P.; Liu, X.; Liang, X.; Zhang, Q.; Wang, Z.; Zheng, Z.; Cheng, H.; Liu, Y.; Dai, Y.; Huang, B. Design and synthesis of $BiVO_4$@CuO_x as a photo assisted Fenton-like catalyst for efficient degradation of tetracycline. *Surf. Interfaces* **2021**, 26, 101380.

[7] Zhao, S.; Chen, C.; Ding, J.; Yang, S.; Zang, Y.; Ren, N. One-pot hydrothermal fabrication of $BiVO_4/Fe_3O_4$/rGO composite photocatalyst for the simulated solar light-driven degradation of rhodamine B. *Front. Environ. Sci. Eng.* **2022**, 16, 36.

[8] Guo, F.; Shi, W.; Lin, X.; Yan, X.; Guo, Y.; Che, G. Novel $BiVO_4/InVO_4$ heterojunctions: Facile synthesis and efficient visible-light photocatalytic performance for the degradation of rhodamine B. *Sep. Purif. Technol.* **2015**, 141, 246-255.

[9] Huang, K.; Hu, T.; Wang, Y. Enhanced photocatalytic degradation of methylene blue through synthesizing of novel of $BiVO_4/Zn_2SnO_4$ under visible light. *J. Solid State Chem.* **2021**, 294, 121864.

[10] Orsetti, F.R.; Bukman, L.; Santos, J.S.; Nagay, B.E.; Rangel, E.C.; Cruz, N.C. Methylene blue and metformin photocatalytic activity of CeO_2-Nb_2O_5 coatings is dependent on the treatment time of plasma electrolytic oxidation on titanium. *Appl. Surf. Sci. Adv.* **2021**, 6, 100143.

[11] Carvalho, K.T.G.; Nogueira, A.E.; Lopes, O.F.; Byzynski, G.; Ribeiro, C. Synthesis of g-C_3N_4/Nb_2O_5 heterostructures and their application on removal of organic pollutants under visible and ultraviolet irradiation. *Ceram. Int.* **2017**, 43, 3521-3530.

[12] Silva, G.T.S.T.; Carvalho, K.T.G.; Lopes, O.F.; Ribeiro, C. g-C_3N_4/Nb_2O_5 heterostructures tailored by sonochemical synthesis: Enhanced photocatalytic performance in oxidation of emerging pollutants driven by visible radiation. *Appl. Catal. B* **2017**, 216, 70-79.

[13] Moraes, N.P.; Bacani, R.; Silva, M.L.C.P.; Campos, T.M.B.; Thim, G.P.; Rodrigues, L.A. Effect of Nb/C ratio in the morphological, structural, optical and photocatalytic properties of novel and inexpensive Nb_2O_5/carbon xerogel composites. *Ceram. Int.* **2018**, 44, 6645-6652.

[14] Hong, Y.; Li, C.; Zhang, G.; Meng, Y.; Yin, B.; Zhao, Y.; Shi, W. Efficient and stable Nb_2O_5 modified g-C_3N_4 photocatalyst for removal of antibiotic pollutant. *Chem. Eng. J.* **2016**, 299, 74-84.

[15] Cui, C.; Guo, R.; Ren, E.; Xiao, H.; Lai, X.; Qin, Q.; Jiang, S.; Shen, H.; Zhou, M.; Qin, W. Facile hydrothermal synthesis of rod-like Nb_2O_5/Nb_2CT_x composites for visible-light driven photocatalytic degradation of organic pollutants. *Environ. Res.* **2021**, 193, 110587.
